# Response to: Comment on “Effect of Exercise Intervention on Flow-Mediated Dilation in Overweight and Obese Adults: Meta-Analysis”

**DOI:** 10.1155/2019/2470581

**Published:** 2019-02-05

**Authors:** Younsun Son, Minsoo Kang, Yoonjung Park

**Affiliations:** ^1^Department of Health & Human Performance, University of Houston, USA; ^2^Department of Health, Exercise Science, and Recreation Management, University of Mississippi, USA

We read with great interest the Letter to the Editor by Mohammad Alwardat [1] on our article [2]. We have addressed each of his concerns as below.

Dr. Alwardat raised the question of using PEDro and recommended using the Grading of Recommendations Assessment, Development and Evaluation (GRADE) approach. We appreciate the opportunity to entertain all other valid options to assess bias risk or methodological quality in individual studies in addition to PEDro, such as Cochrane's Collaboration of risk of bias [3], Newcastle-Ottawa Scale [4], and Down and Black checklist [5]. We also value the potential use of the GRADE system that Dr. Alwardat recommended, yet the GRADE system is used for grading the quality of “body of evidence” rather than the study level [6]. It also does not provide a quantitative rating of bias risk. We elected to display the PEDro scores of the articles in our data as we believe it will facilitate the reader's understanding of the relative level of quality of evidence contained therein, which the GRADE system was not able to provide. In addition, Dr. Alwardat mentioned that in his opinion PEDro values of 6 were relatively low for our type of study; however, the reference he cited [7] examined risk of bias methods specifically when using PEDro cutoff scores, which we did not use in our study. Furthermore, studies with PEDro scores of 6 or higher are widely ascribed terms such as to be of “good”, “fair”, or “moderate” [8–10], and thus our choice of language to describe our results is justified. As an additional clarification, we would like to inform readers that our median PEDro scores for included studies are 8 with an interquartile range (IQR) of 3. Over 76% of the included studies have the PEDro score of 6 or higher.

Another comment referred to our inclusion criteria. It was suggested that our inclusion criteria were ambiguous and did not follow the PICO (P: participants, I: intervention, C: comparison, O: outcomes) format; however, it is outlined clearly in our paper in the Methods section. Indeed, the commenter's own written response claims the study is lacking defined “outcome measures” while quoting in the same paragraph a section of our paper which includes a description of exactly such an included outcome measure: “studies included the value of relative flow-mediated dilation…”. It was also suggested that our study lacked “comparison” groups, but this is again inaccurate; comparisons such as exercise duration, modality, and intensity (among others) can be clearly seen in the figure and tables and in our moderator analysis results.

The final observation pertains to the use of MeSH (Medical Subject Headings) in addition to keywords in the search of related articles when constructing a meta-analysis. We appreciate the opportunity to clarify the details of our methodology and understand that searching using both free text and MeSH is an important aspect of meta-analysis methodology; failing to do so would risk missing relevant articles. We did perform such a search, using both keywords and MeSH as appropriate, when obtaining relevant articles for our study using PubMed. We did not feel this particular detail of our methodology bore special delineation in our Methods section, and thus it was not specifically mentioned that we had done so. We thank Dr. Alwardat for the chance to provide this detail to our readers.
